# The Extent to Which Different Executive Function Skills Mediate the Spatial-Math Link

**DOI:** 10.3390/bs16091545

**Published:** 2026-09-01

**Authors:** Elyssa A. Geer, Brianna L. Devlin, Tracy M. Zehner, Irem Korucu, Lindsey Bryant, David J. Purpura, Robert J. Duncan, Sara A. Schmitt

**Affiliations:** 1 Anita Zucker Center for the Excellence in Early Childhood Studies, University of Florida, Gainesville, FL 32601, USA; 2 Department of Learning and Instruction, The State University of New York at Buffalo, Buffalo, NY 14260, USA; bdevlin@buffalo.edu; 3 Prevention Science Institute, University of Oregon, 1600 Millrace Dr., Eugene, OR 97403, USA; tzehner@uoregon.edu (T.M.Z.); sschmitt@uoregon.edu (S.A.S.); 4 Department of Psychology, Florida Atlantic University, Boca Raton, FL 33431, USA; ikorucu@fau.edu; 5 Department of Human Development and Family Science, Purdue University, West Lafayette, IN 47907, USA; bryant77@alumni.purdue.edu (L.B.); purpura@purdue.edu (D.J.P.); 6 Department of Human Development and Family Studies, Colorado State University, Fort Collins, CO 80523, USA; rob.duncan@colostate.edu; 7 Department of Special Education and Clinical Sciences, University of Oregon, 1655 Alder Street, Eugene, OR 97401, USA

**Keywords:** spatial skills, mathematics, executive function, preschool, mediation

## Abstract

Understanding factors related to early math development is critically important because early math skills predict a number of academic outcomes, such as later math, reading, and school success. Links between spatial and math skills and between executive function and math skills have been established consistently throughout extant research across a variety of ages and skill types. However, little work has examined how these three skillsets are connected; specifically, whether executive function mediates the spatial-math relation. Although past research has found no mediation, this research only included three measures of executive function skills and treated executive function as a single construct, rather than examining the potentially different roles of various executive function skills in the relationship between spatial and math skills. The present study addressed this gap by examining whether five executive function skills (i.e., working memory, inhibitory control, cognitive flexibility, behavioral self-regulation, planning) mediate the relationship between spatial and math skills in preschoolers. Results showed that cognitive flexibility and behavioral self-regulation uniquely mediated this relation, providing preliminary evidence that three specific executive functions (cognitive flexibility, behavioral self-regulation, and planning) mediate the relationship between spatial and math skills. Implications and future directions are discussed.

## 1. Introduction

Understanding how spatial skills and executive functions are related to early math development has far-reaching implications for understanding factors that may impact later academic achievement (e.g., Claessens & Engel, 2013; Jordan et al., 2009; Purpura et al., 2017; Ten Braak et al., 2022). A substantial body of research has documented consistent associations between spatial skills and mathematics (for a meta-analytic review, see Atit et al., 2022) and between executive function and mathematics (e.g., Ellis et al., 2021; Mazzocco & Kover, 2007; Ribner et al., 2017; Schmitt et al., 2017) throughout development. Research on these relationships has demonstrated that stronger spatial skills and executive function skills, separately, are predictive of better math skills and that these relationships exist as early as preschool (Rittle-Johnson et al., 2019; Verdine et al., 2017). Although connections between spatial and math skills as well as executive function and math are well-established in research, it remains unclear how these three skillsets may be interconnected, particularly in young children. It is possible that executive functions may underlie the spatial-math link due to shared cognitive capacities that are essential to each of these skills.

Recently, research has begun to examine the role of executive function as a possible mediator in the spatial-math link in children (Hawes et al., 2019). Hawes and colleagues found that executive function did not mediate the relationship between spatial and math skills in a sample of 4- to 11-year-old children. Hawes et al. (2019) examined executive function as a latent construct derived from multiple executive function measures (two working memory and one behavioral self-regulation), whereas the present study focuses on whether individual executive function components differentially account for the spatial–mathematics association. While there is some disagreement between proposed models of executive function that conceptualize executive function unidimensionally versus multidimensionally, Ibbotson suggested that executive function develops in a hierarchy, with early executive function being unidimensional early in development and becoming more multidimensional with age and experience. However, extant research in preschoolers has demonstrated that individual components of executive function differentially relate to math skills (Purpura et al., 2017), suggesting that it may be beneficial to examine the role of individual components of executive function skills in the spatial-math link. Although Hawes and colleagues have provided important contributions to the field by examining whether a unidimensional executive function factor served as a mediator of the spatial-math link in 4- to 11-year-old children, we posit that it remains unclear whether individual components of executive function may mediate the spatial-math link in early childhood because of the potential unique relationship between individual aspects of executive function and mathematics. Thus, the goal of the present study is to address a different research question by examining if individual executive function skills mediate the relationship between spatial and math skills in preschoolers.

### 1.1. The Spatial-Math Link

Spatial skills refer to a broad set of cognitive abilities that facilitate successful interaction with and navigation of the spatial world, supporting a variety of everyday tasks such as driving, parallel parking, packing a suitcase, and arranging furniture (Linn & Petersen, 1985; Uttal et al., 2013; Vasilyeva & Lourenco, 2010). Across the lifespan, research has found assorted spatial skills to be consistent predictors of math outcomes (Casey et al., 1995, 2001; Delgado & Prieto, 2004; Geer et al., 2019, 2025; Shea et al., 2001; Uttal et al., 2013) and despite the consistent link between various spatial and math skills, these skillsets are considered to be related but distinct (Mix et al., 2016). Consistent with this, a recent meta-analysis confirmed the spatial-math association across a variety of tasks and ages (Atit et al., 2022). Walsh’s (2003) Theory of Magnitude (ATOM) proposes that spatial and numerical abilities develop from a shared magnitude-processing system, primarily supported by the parietal cortex, providing one explanation for the consistent association between spatial skills and mathematics achievement (Walsh, 2003). The relationship between spatial and math skills has been shown to emerge early in childhood, with extant research establishing the link across various subtypes of spatial skills and math (Geer et al., 2019, 2025; Verdine et al., 2017).

Some existing research has directly linked early spatial skills to specific early math skills such as numeracy in early childhood (Geer et al., 2025; Rittle-Johnson et al., 2019). Numeracy, the focus of the current study, involves math skills that do not require written mathematical symbols (e.g., verbal counting, cardinality) that can be learned in everyday environments (e.g., play; Baroody et al., 1984; Purpura & Lonigan, 2013). Because aspects of both early spatial skills and early numeracy skills can be learned during informal activities such as play, these skills form an important foundation for more complicated spatial and math skills that are acquired later in childhood (Eason & Ramani, 2020; Libertus et al., 2013; Purpura & Lonigan, 2013; Zippert et al., 2019).

Although substantial evidence supports an association between early spatial skills and mathematics, less work has explored *how* these skills are connected. One possibility is that spatial and math skills may be connected *indirectly* through more domain-general cognitive skills (such as executive functions). This may be particularly true for young children, whose understanding of early spatial and math experiences may stem from underlying cognitive capacities that facilitate success on spatial tasks and the development of early mathematical understanding. Executive function has been identified as a common correlate of both early spatial and mathematical skills and may represent an important mechanism underlying the association between these domains (Fung et al., 2020; Geer et al., 2025; Hawes et al., 2019; Kahl et al., 2021, 2022; Verdine et al., 2014b).

### 1.2. Executive Function

Executive function encompasses a broad skillset that is flexible and supports the engagement of goal-directed behaviors (Garon et al., 2008; Lezak, 1995; Mesulam, 2002). Drawing on Miyake et al. (2001) Unity-and-Diversity Model, executive function can be operationalized as a multidimensional construct comprising inhibitory control, working memory, and cognitive flexibility, which are distinguishable yet interrelated components of cognitive control. Working memory is the ability to encode information, carry it in the mind, and utilize it in the successful completion of a goal (e.g., the ability for a child to listen to a word problem, hold key information in their mind, and later use that information to solve the problem; Garon et al., 2008; Schmitt et al., 2018). Inhibitory control is the ability to suppress a prepotent or inappropriate response in favor of a more adaptive one (e.g., a child inhibiting the use of an incorrect or less effective strategy when solving a mathematics problem in order to apply a more appropriate solution strategy; Garon et al., 2008; Schmitt et al., 2018). Cognitive flexibility is the ability to adjust one’s approach and shift attention between changing tasks, contexts, or goals (e.g., adapting to different representations of quantity by shifting from visual dot patterns to numerical symbols; Garon et al., 2008; Schmitt et al., 2018).

Working memory, cognitive flexibility, and inhibitory control are considered to be more *simple*, laying the groundwork for more complex executive function skills (Best & Miller, 2010; Diamond, 2013; Garon et al., 2008; Vaidya et al., 2020). Consistent with this, Diamond’s (2013) developmental framework suggests that these three executive functions are the core executive functions and serve as foundational skills that enable more complex cognitive abilities such as planning, reasoning, and problem solving. Complex executive functions emerge through the coordination and integration of more foundational executive processes (Jones et al., 2016). Behavioral self-regulation is one such multifaceted executive function that reflects children’s ability to integrate working memory, inhibitory control, and cognitive flexibility to regulate their behavior in accordance with task demands and goals (Ponitz et al., 2009). For example, the ability to participate in a game of “Simon Says” in which you have to learn and remember the cardinal rule of only doing what “Simon Says”, while controlling any undesired behaviors *and* flexibly shifting between new tasks that you must do because “Simon Says” is a real-world example of behavioral self-regulation. Planning is another complex executive function that involves organizing and coordinating thoughts and actions across multiple steps to successfully achieve a goal (Allain et al., 2005). For example, a child engages their planning skills when they plan out a structure that they want to build using blocks in their classroom (e.g., a castle) and take the necessary steps to build such a structure (e.g., using bigger blocks at the base to make a foundation, then progressively smaller blocks to make the castle walls and towers).

In preschool-aged children, executive function can be conceptualized as a set of emerging cognitive processes that support goal-directed behavior, self-regulation, and adaptive responding to environmental demands (Garon et al., 2008). In line with this, Diamond (2013) suggests that the core executive functions (working memory, cognitive flexibility, and inhibitory control) form a foundation for complex executive functions (such as planning), which suggests the core executive functions emerge first and directly contribute to the development of more complex executive functions. Preschoolers’ executive function skills are further reflected in children’s ability to represent, maintain, and flexibly apply increasingly complex rule structures, particularly when required to switch between competing task demands (Zelazo et al., 2003). Accordingly, executive function in preschoolers may be operationalized through performance on developmentally appropriate tasks assessing various aspects of executive function, as these abilities collectively reflect children’s emerging capacity for cognitive control and self-regulated behavior.

While there is some debate about whether executive function is unidimensional and multidimensional, Ibbotson (2023) argues that the apparent inconsistency between unidimensional and multidimensional models of executive function reflects developmental change rather than competing theories. Executive function is relatively unified during early childhood but becomes increasingly differentiated as children acquire experience and develop more specialized cognitive control processes. Thus, executive function is best conceptualized as a dynamic, progressively differentiating system rather than either exclusively unitary or multidimensional. Importantly, regardless of the unidimensional or multidimensional view of executive function, extant research in preschoolers has demonstrated that various executive function skills differentially predict math skills (Purpura et al., 2017); therefore, the present study considers how individual components of executive function may differentially relate to the spatial-math link in preschoolers rather than creating one unidimensional factor for executive function.

### 1.3. Executive Function and the Spatial-Math Link

Connections between spatial skills and executive function as well as executive function and math skills *separately* have been established in children as young as preschool, with research showing stronger spatial skills predicting stronger executive function skills as well as stronger executive function skills predicting strong spatial and math skills (spatial predicting executive function, Kahl et al., 2022; executive function predicting spatial, Geer et al., 2025; Hawes et al., 2019; Kahl et al., 2021, 2022; executive function predicting math, Geer et al., 2025; Ribner et al., 2017; Schmitt et al., 2017; Verdine et al., 2014b). It is of note that much of the extant research on the relationships between spatial and executive function skills is examined concurrently (i.e., Garcia et al., 2022; Geer et al., 2025; Hawes et al., 2019; He et al., 2019). Based on the existing research examining spatial skills as a predictor of executive function (Kahl et al., 2022) as well as research that has identified spatial and executive function skills as potentially confounding variables that represent unique but highly intertwined domain-general skills (Hawes et al., 2019), it is necessary to examine the potential interplay between spatial and executive function skills more closely. Some extant research has begun to explore the interplay between spatial, math, and executive function skills, specifically, expanding what we know about the relationships between all three of these skillsets (Atit et al., 2022; Fung et al., 2020; Geer et al., 2025; Hawes et al., 2019; Kahl et al., 2021, 2022; Verdine et al., 2014b; Yang et al., 2019). From this work, it has become clear that spatial, math, and executive function skills are interconnected, but the role executive function may play in the spatial-math link remains unclear.

Although reciprocal relationships among spatial skills, executive function, and mathematics are plausible from a developmental perspective (Geer et al., 2019; Diamond, 2013), the present study focuses on one theoretically motivated pathway: whether earlier spatial skills contribute to later mathematical performance in part through the development of executive function. Testing this pathway does not preclude alternative reciprocal influences but instead evaluates one candidate mechanism that has received comparatively little attention. Past research has considered executive function as a mediator between other cognitive factors such as early math and later math and reading (Ten Braak et al., 2022), due to the practice of early math serving to strengthen executive function skills that are necessary for success in later math and reading. This suggests that it is worth considering that executive function may mediate the link between spatial and math skills, though most work neglects this possibility. One exception is a study by Hawes et al. (2019), where they explored the interplay between four factors (i.e., numerical, spatial, mathematics, and executive function skills). Hawes and colleagues examined the extent to which a unidimensional latent factor of executive function mediated the relationship between spatial and math skills. They hypothesized that spatial and math skills are related due to the shared executive function demands that underly each of these skillsets, and they designed their model in order to test that hypothesis. Their results did not indicate that executive function significantly mediated the spatial-math link. Hawes and colleagues interpreted this non-significant mediation as evidence that spatial skills and executive function differentially relate to math achievement, with spatial skills having a more direct link to math, which is in line with extant research that has demonstrated that spatial skills uniquely predicted math, even when holding differences in executive function constant (Verdine et al., 2014b).

Another possibility, however, is to consider executive function as multidimensional, with specific executive function skills possibly relating differently to the spatial-math association. This contention is supported by work that has demonstrated specific executive functions relate to different mathematical outcomes in preschoolers (Purpura et al., 2017). Furthermore, while the Hawes et al. (2019) study provided valuable answers regarding the potential role of executive function as a unitary latent factor, they included only measures of working memory and behavioral self-regulation. It is possible that including other measures of executive function may alter the pattern of results. We posit that it is possible that when tested separately, working memory or behavioral self-regulation may explain part or all of the spatial-math link. Similarly, other types of executive function (e.g., cognitive flexibility, inhibitory control, planning) may explain part or all of the spatial-math link, though no research has examined this possibility.

Although no existing research has examined specific executive function skills as possibly explaining part or all of the connection between spatial and math skills, there is some work that provides a preliminary understanding of the interplay between spatial, math, and specific executive function skills, and this research suggests that cognitive flexibility may be particularly important (Kahl et al., 2022). For example, Kahl et al. (2022) ran a cross-lagged panel model to examine potential bidirectional relationships between spatial, math, and executive function skills (specifically, they measured working memory, inhibitory control, and cognitive flexibility skills) in primary school children. They found that spatial visualization skills predicted cognitive flexibility but not working memory or inhibitory control (when accounting for previous levels of these skills). These results suggest that strong spatial visualization skills, which involve the ability to solve complicated multi-step problems using spatial-presented stimuli (Linn & Petersen, 1985), may lead to improved cognitive flexibility skills because these tasks often require the child to think flexibly about how to accurately solve each item (i.e., being able to quickly adapt/change their strategy for task completion from item to item). Importantly, although Kahl et al. (2022) did not find that cognitive flexibility significantly predicted math achievement, other existing research has established cognitive flexibility as a significant predictor of math skills in children (Geer et al., 2025; Magalhães et al., 2020). Examining specific executive functions as potential mediators of the spatial-math relation, rather than creating a latent factor or average executive function score, is important because it will allow us to determine potential unique associations between individual executive function skills and the relationship between spatial and math skills.

Despite the lack of empirical evidence directly testing if specific executive functions underlie or explain the link between spatial and math skills, it is reasonable to conclude that various executive functions are unlikely to contribute uniformly to the relationship between spatial and mathematical skills because individual components of executive function support different cognitive operations. Most spatial tasks require maintaining and manipulating spatial representations, suppressing irrelevant visual information, and flexibly shifting between multiple representations or solution strategies. And mathematical problem solving similarly relies on maintaining intermediate results, inhibiting impulsive or incorrect responses, and coordinating multiple procedures. Consequently, different executive function skills may support distinct aspects of the spatial–mathematics relationship rather than functioning as a single, unitary mechanism. Rather than assuming that executive function operates as a single, unitary mechanism, the present study examines whether individual executive function processes (i.e., working memory, inhibitory control, cognitive flexibility, behavioral self-regulation, planning) differentially account for the association between spatial and math skills.

Working memory may be particularly important because both spatial and mathematical tasks require the temporary maintenance and manipulation of information. Solving spatial problems often involves mentally transforming or updating representations, whereas mathematical problem solving requires holding intermediate results and coordinating multiple pieces of information. Inhibitory control may facilitate both spatial and mathematical performance by suppressing distracting information, preventing incorrect responses, and reducing interference from competing solution strategies. Cognitive flexibility may support performance by allowing children to shift between representations, adapt strategies when problems become more complex, and integrate spatial and symbolic information. Behavioral self-regulation may influence the application of spatial and executive skills during mathematics by supporting sustained attention, persistence, and goal-directed behavior throughout complex learning activities and problem-solving. Complex planning may be particularly relevant because many spatial and mathematical tasks require children to anticipate multiple steps, organize sequences of actions, and evaluate progress toward a solution rather than relying on a single cognitive operation. Because these executive processes support partially distinct cognitive operations, examining them separately may provide greater insight into the mechanisms linking spatial and mathematical skills than treating executive function as a single latent construct.

### 1.4. Present Study

The present study aims to establish preliminary evidence about whether distinct executive function skills mediate the association between spatial and mathematical skills across two waves of data collected from preschool children, primarily from low-income backgrounds. Our analyses are largely exploratory given that this is the first study to examine whether specific executive functions mediate the spatial-math link; however, based on existing research, there is a strong likelihood that cognitive flexibility may mediate the spatial-math link, as it has been established that spatial skills predict cognitive flexibility (Kahl et al., 2022) and cognitive flexibility predicts math (Geer et al., 2025; Magalhães et al., 2020).

## 2. Methods

### 2.1. Participants

Participants in this study were 242 preschoolers (*M_age_* = 52.01 months, *SD_age_* = 6.78 months) from two data collection sites in the midwestern United States. All participants came from families with low incomes, as defined by Head Start program status (*n* = 66) or eligibility for free/reduced-price lunch (*n* = 176). The sample was split fairly evenly by sex, with 49% of the sample being reported by parents as being female (120 female, 122 male). Participants’ racial/ethnic background was reported by parents, with 33.1% identifying as Latinx/Hispanic, 32.2% identifying as White, 13.6% identifying as Multiracial, 9.9% identifying as Asian, 6.2% identifying as Black/African American, 0.8% identifying as Other, and 4.2% who did not answer this question. A portion of our sample (19.8%) were English Language Learners (ELL) and completed the assessments in Spanish. On average, participants’ parents had a high school diploma. Specifically, 2.1% of our sample reported 8th grade or less, 12% reported some high school, 7% reported General Equivalency Diploma (GED), 24% reported high school diploma, 20.7% reported some college, 7% reported associate’s degree, 13.2% reported bachelor’s degree, 6.2% reported master’s degree, and 0.8% reported doctoral degree. Seven percent of the sample did not report education level.

### 2.2. Missing Data

Data used for this project were collected during fall 2021 (pretest) and spring 2022 (posttest) as part of a larger intervention study (Schmitt et al., 2025). It should be noted that prior to the intervention, 12 children dropped out of the study for a variety of reasons, but they did not significantly differ on any demographic characteristics from children who remained in the study.

### 2.3. Measures

#### 2.3.1. Spatial Skills

To assess 2-dimensional and 3-dimensional spatial assembly skills, we administered the Test of Spatial Assembly (TOSA; Verdine et al., 2014a, 2014b, 2017). For this measure, participants are required to use provided models to build a specific design using 2- (shapes on laminated paper) and 3-dimensional objects (LEGO^®^ bricks). The 2-dimensional portion of the task consists of seven items (one practice item and six scored items). For each item, participants are presented with a 2-dimensional model composed of two to four uniquely shaped pieces (e.g., circles, squares) and are instructed to arrange their own pieces to match the provided model. Performance is scored using a composite score that captures three components of accuracy: adjacent piece placement, horizontal and vertical orientation, and relative position. Item-level composite scores range from 0 to 10 points, with higher scores reflecting more complex models, resulting in a maximum possible total score of 35 points. The 3-dimensional portion of the task consists of seven items (one practice item and six scored items). For each item, participants are presented with a LEGO^®^ structure ranging in complexity from two to four pieces across varying configurations and are instructed to recreate the model using their own LEGO^®^ bricks. Performance on each item is scored using a composite score that captures three components of spatial accuracy: vertical location, rotation, and translation. For models containing three or more LEGO^®^ pieces, composite scores were also calculated for each possible two-piece subset within the model using the same three components. Scores for individual models range from 0 to 8 points, depending on model complexity, whereas scores for two-piece subsets range from 0 to 3 points. The total composite score for the 3-dimensional portion of the task is 46 points. Detailed information regarding task administration and scoring procedures can be found in Verdine and Golinkoff (2018). The TOSA demonstrated high internal consistency across both waves of data collection (pretest α = 0.87; posttest α = 0.85).

#### 2.3.2. Numeracy Skills

To measure early numeracy skills, we administered the Preschool Early Numeracy Skills task (PENS; Purpura et al., 2015). This measure is designed to assess a broad range of numeracy skills that children are expected to develop during the preschool and kindergarten years. The PENS consists of 24 items assessing multiple aspects of early mathematics knowledge, including numerical identification, number order, ordinality, one-to-one correspondence, numerical comparison, set comparison, and number combinations. Items vary in format, with some requiring multiple-choice responses and others requiring open-ended responses. The assessment includes a discontinuation rule after three consecutive incorrect responses. Total scores are calculated by summing performance across items, with correct responses receiving 1 point and missing or incorrect responses receiving 0 points. The PENS has high internal consistency across both time points (pretest α = 0.91; posttest α = 0.93).

#### 2.3.3. Executive Function Skills

**Working Memory.** To measure working memory, we administered the Hide and Seek task (HAS; Garon et al., 2014). For this task, participants are presented with a set of four boxes arranged in a vertical 2 × 2 square with flaps that open towards the participant. For each trial, a foam block is hidden inside a different box. After the block is hidden, the set of four boxes is covered with a white cloth and 10 s elapse. The researcher then uncovers the boxes and asks the participant to indicate the location of the hidden block. Participants have up to four attempts to locate the block on each trial; if the block is not found after the fourth attempt, the participant receives a score of 0 for that item. Item scores range from 0, indicating that the participant was unable to locate the block, to 4, indicating that the participant identified the correct location on the first attempt. Total scores are calculated by summing scores across items, with higher scores reflecting better performance, as fewer attempts were required to locate the block. The task demonstrated acceptable internal consistency at both time points (pretest α = 0.77; posttest α = 0.75).

**Inhibitory Control.** To measure inhibitory control, we administered the Day-Night Stroop task (DNS; Gerstadt et al., 1994). For this task, participants are instructed to provide the opposite response of what is depicted on each card. Specifically, when presented with a card showing the moon and stars (i.e., night), participants are instructed to respond “day,” whereas when presented with a card showing the sun (i.e., day), they are instructed to respond “night.” The task includes two practice trials to ensure that participants understand the instructions. Practice trials may be repeated up to three times if the participant demonstrates confusion or does not understand the task requirements. The participant then receives 14 testing items. For each item (both trial and testing items), scores range from a 0 for incorrect to a 1 for a similar word (i.e., sun instead of day)/self-correction to a 2 for a correct response. These scores are then summed, such that total scores on this task range from 0 to 32. The test has high internal consistency at both time points (pretest α = 0.90; posttest α = 0.89).

**Cognitive Flexibility.** To assess cognitive flexibility, we administered the Card Sorting Task, which is adapted from the Dimensional Change Card Sort (Zelazo, 2006). In this task, participants sort cards depicting animals (e.g., dog, fish, bird) that vary in color (e.g., red, blue, yellow) and size (e.g., small, medium, large) into one of four boxes (large blue dog, small red fish, medium yellow bird, and a green frog foil). The task consists of four parts, each containing six items. In the first part, participants sort cards by shape while ignoring color and size. In the second part, participants sort cards by color while ignoring shape and size. In the third part, participants sort cards by size while ignoring shape and color. Participants who score 5 or higher on the third part (1 point per correct response) proceed to the fourth part, which requires participants to flexibly switch sorting rules by sorting cards by size when the image includes a black border and by color when the image does not include a border. Total scores are calculated by summing correct responses across all four parts, with scores ranging from 0 to 24 points. The task demonstrated high internal consistency across both time points (pretest α = 0.93; posttest α = 0.95).

**Behavioral Self-Regulation.** To assess behavioral self-regulation, we administered the Head-Toes-Knees-Shoulders task (HTKS; McClelland & Cameron, 2011). The HTKS consists of three parts, each containing 10 items, that progressively increase in difficulty by requiring participants to inhibit a prepotent response and apply increasingly complex rules. In the first part, participants are instructed to perform the opposite action of the researcher’s prompt using two response options: head and toes. For example, when instructed to “touch your head,” participants should touch their toes. Participants receive 2 points for each correct response, 1 point for a self-corrected response, and 0 points for an incorrect response, resulting in a possible score of 20 points. Participants who achieve at least 4 points on the first part proceed to the second part, which follows the same procedure but uses shoulders and knees as the response options (e.g., touching shoulders when instructed to touch knees). Participants who achieve at least 4 points on the second part proceed to the third part, which requires integrating and switching between the rules from the previous parts while also incorporating new body-part pairings (e.g., head-knees and toes-shoulders). For example, when instructed to “touch your toes,” participants must touch their shoulders, which conflicts with the response mappings established in earlier parts. Total scores are calculated by summing performance across all items, with scores ranging from 0 to 60 points, where higher scores indicate stronger behavioral self-regulation. The task demonstrated high internal consistency across both time points (pretest α = 0.98; posttest α = 0.98).

**Planning.** To measure planning skills, we administered the Tower of Hanoi task (TOH; Carlson et al., 2004; Welsh, 1991). This is a simplified version of the original task, developed by Simon (1975), and is more appropriate for young children. For this version of the task, participants were introduced to the activity as a game in which the wooden disks represented monkeys (the smallest disk as the baby monkey, the medium-sized disk as the little monkey, and the largest disk as the big monkey) and the three posts represented trees. The participant and researcher each had identical sets of monkeys and trees, allowing the child to reproduce the researcher’s model for each level. The task consisted of six trials, with each trial allowing up to two attempts. If a participant was unable to complete a level on the first attempt, the researcher repeated the trial; if the participant was unsuccessful on the second attempt, the task was discontinued. Difficulty increased incrementally across levels. Specifically, level one required participants to move two disks in a single move to match the researcher’s model; level two required two moves with two disks; level three required three moves with two disks; level four required two moves with three disks; level five required three moves with three disks; and level six required four moves with three disks. Scores on this task range from 0 to 6. The test has acceptable internal consistency across both time points (pretest α = 0.69; posttest α = 0.59).

### 2.4. Covariates

Condition. Participants were randomly sorted into one of three conditions: business-as-usual (control), free-play block play condition, and semi-structured block play condition. The intervention condition that the child was in was included in all analyses to adjust for any intervention effects on the included variables between pretest and posttest. Notably, although patterns of positive effects were found in the original intervention study on the outcomes included in the current study, none of these effects were significant (Schmitt et al., 2025).

Sex. Parents reported their child’s sex prior to participation through a brief demographic survey. They were asked to provide their child’s biological sex using an open-response format. Given evidence of sex differences in mathematics performance across a range of ages and tasks (Ganley & Vasilyeva, 2011; Halpern et al., 2007; Lachance & Mazzocco, 2006; Meece et al., 1982), sex was included as a covariate in all analyses.

Age. Age was calculated based on each participant’s date of birth, which was provided by parents during the brief demographic survey. Because older children may demonstrate stronger mathematics skills due to increased opportunities for learning and experience (Aunola et al., 2004), age at pretest was included as a covariate in all analyses.

Race/ethnicity. Parents reported their child’s race/ethnicity as part of the brief demographic survey. Parents selected from the following categories: Asian, Black/African American, Latinx/Hispanic, White, Multiracial, or Other, with an open-response option provided for additional information. Given evidence that mathematics outcomes may differ across racial and ethnic groups (Riegle-Crumb & Grodsky, 2010), race/ethnicity was included as a covariate in all analyses.

**Parent education.** Parents reported their education level during the brief demographic survey. For this, there was a question for each of the caregivers, asking their educational level (i.e., 8th grade or less, some high school, General Equivalency Diploma (GED), high school diploma, some college, associate’s degree, bachelor’s degree, master’s degree, and doctoral degree). If information was provided for only one caregiver, parent education was coded based on that caregiver’s highest level of education. If information was provided for both caregivers, parent education was coded as the highest level of education reported across caregivers (e.g., if one caregiver had a bachelor’s degree and the other had completed a GED, the family’s parent education variable was classified as a bachelor’s degree). Parent education was included as a covariate in all analyses given evidence that higher levels of parent education are associated with stronger early mathematics skills (Slusser et al., 2019).

**ELL status.** Parents reported their child’s primary language as English, Spanish, or Other (with an option to provide an additional response). Children whose primary language was reported as Spanish were classified as English language learners (ELLs). ELL status was included as a covariate in all analyses because ELL participants completed all study assessments in Spanish, which were designed to parallel the English versions. Although the assessments were intended to be comparable across languages, accounting for ELL status helps address potential differences between language versions that could influence performance.

**Picture Vocabulary Test**. To account for receptive vocabulary skills, we administered the Toolbox Picture Vocabulary Test (PVT; Hodes et al., 2013). This assessment is administered on a tablet through the NIH Toolbox application (Hodes et al., 2013), in which participants are presented with four images of different objects and asked to identify the image that corresponds to a spoken word prompt (e.g., “touch banana!”). The task includes several practice items during which the program provides verbal feedback (e.g., “that’s right!” or “try again”) to ensure participants understand the instructions. Consistent with our other assessments, the PVT was administered in Spanish for all English language learners (ELLs) in the study to minimize the influence of limited English proficiency on performance; the Spanish version has been validated by Gershon et al. (2020). The NIH Toolbox application generates total PVT scores using item response theory (IRT), producing a theta score for each participant that reflects overall performance and is normed by age. This theta score is analogous to a standardized score, with a mean of zero and a standard deviation of one. Pretest PVT scores were included as a covariate in all analyses to account for individual differences in receptive vocabulary, which may influence children’s ability to understand verbal instructions across study tasks.

### 2.5. Procedure

Participants were recruited through preschool programs that had already agreed to be a part of this research project. During fall 2021, which was the pretest time point for the larger intervention study, participants completed a series of tasks in one of four randomized orders to mitigate any effects of task order. Tasks were given in one-on-one sessions by trained research assistants in classrooms or break rooms as designated by each school. Participants were tested in 3 to 4 testing sessions that lasted approximately 20–30 min each due to the length of the testing battery. Following pretesting, participants were assigned to one of three intervention conditions (business-as-usual, free-play block play condition, and a semi-structured block play condition at the classroom level). The intervention began in early spring 2022, and participants who were assigned to either block play condition (free play or semi-structured) engaged in sixteen 15-min small group block play sessions over eight weeks. Following the completion of the intervention, posttest assessment began later in spring 2022. Posttest procedures were identical to pretest.

### 2.6. Data Analysis Plan

All analyses were conducted using MPlus (Muthén & Muthén, 1998–2017) and Stata software (Stata version 18; StataCorp LLC, 2017). We assessed the data for homogeneity of variance and normality prior to the analysis. Additionally, Little’s MCAR test was conducted in Stata (StataCorp LLC, 2017) to determine if data were missing completely at random (MCAR). The statistic was non-significant (*χ*^2^ [132, N = 242] = 133.06, *p* = 0.482), suggesting that data were MCAR. We employed full information maximum likelihood (FIML) to handle missing data and reduce the possibility of obtaining biased estimates during analyses (Acock, 2013). Prior to analyses, all continuous variables were centered to avoid issues with multicollinearity and to aid in interpretability of the results. To examine how different executive function skills may mediate the relationship between early spatial assembly skills and later numeracy skills, we ran a series of mediation analyses. Specifically, each model had pretest spatial skills predicting posttest numeracy skills with a mean score for each individual executive function as a mediator of this relation. Additionally, each model included pretest numeracy, intervention condition, age, sex, race/ethnicity, ELL status, and pretest vocabulary skill as controls (Models 1a–1e). Pretest numeracy was included specifically to control the autoregressive pathway for numeracy, to ensure we controlled for everything that influences posttest outcomes. Thus, allowing us to more effectively examine how children are changing during the period of study above and beyond past levels of numeracy. For these models, we computed a mean score across pretest and posttest scores for each measure of executive function to be used as the mediation term. This was done following statistical techniques used by King and Purpura (2021). Specifically, King and Purpura did this because while it is recommended to use three separate time points or waves of data when running mediation analyses with longitudinal data (Cole & Maxwell, 2003), they did not have three waves of data. Similarly, we do not have three waves of data in our study; as such, each executive function average score was used (following King & Purpura, 2021) because it provided a better estimate of children’s executive function skills between pretest and posttest (National Institute of Child Health and Human Development Early Child Care Research Network & Duncan, 2003). Finally, we conducted a path analysis model including all executive function skills that emerged as significant mediators in individual analyses (i.e., cognitive flexibility, behavioral self-regulation, and planning) as mediators of the relationship between children’s spatial and numeracy skills to examine potentially unique associations between each executive function and the spatial-math link (Model 2).

## 3. Results

### 3.1. Descriptive Statistics

We present all descriptive statistics in Table 1. The variables are normally distributed with no significant skew or kurtosis (Hair et al., 2010). Zero-order correlations between all measures at pretest and posttest can be found in Table 2. We found significant relationships between all included measures of spatial, executive function, and math skills at pretest, except for working memory and spatial skills (*r* = 0.11, *p* = 0.096), working memory and inhibitory control (*r* = 0.09, *p* = 0.157), working memory and cognitive flexibility (*r* = 0.10, *p* = 0.138), and working memory and behavioral self-regulation (*r* = 0.13, *p* = 0.058). Posttest, we found significant correlations between all measures of spatial, executive function, and math skills except for posttest working memory and spatial skills (*r* = 0.13, *p* = 0.070). We also found significant correlations between all measures of spatial, executive function, and math skills across pre- and posttest except for pretest working memory and posttest spatial skills (*r* = 0.04, *p* = 0.551), pretest working memory and posttest math skills (*r* = 0.11, *p* = 0.105), pretest working memory and posttest working memory (*r* = 0.12, *p* = 0.079), and pretest working memory and pretest inhibitory control (*r* = 0.09, *p* = 0.208).

### 3.2. Executive Function as a Mediator of the Spatial-Math Link

Detailed results for all mediation analyses can be found in Table 3 and Table 4. For the sake of clarity, we focus below on the results that are relevant to our research questions.

#### 3.2.1. Model 1a—Working Memory

**Figure 1:**
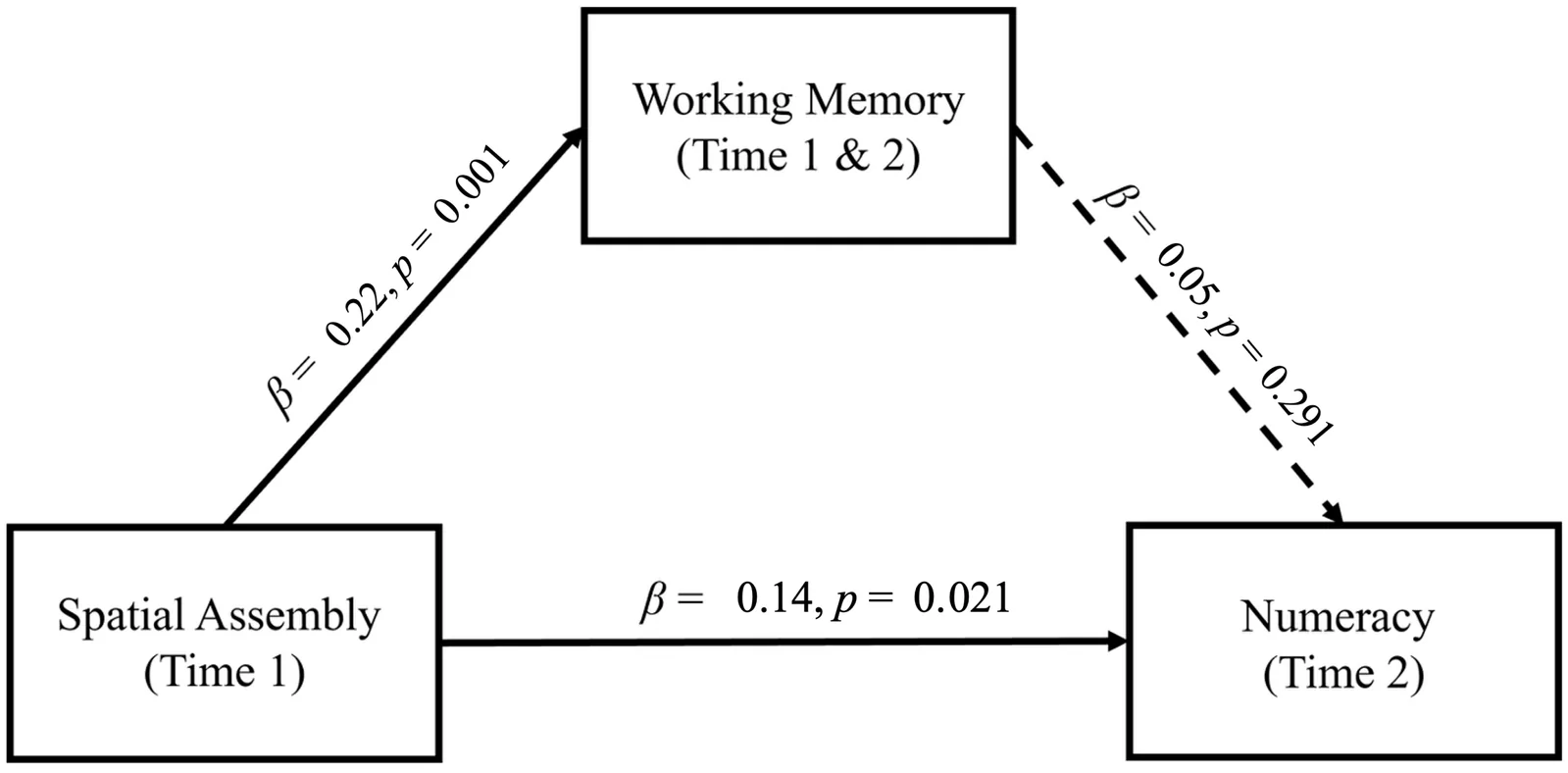
Working Memory as a Mediator of the Spatial-Math Link. *Note.* Working Memory refers to a mean score across pre- and posttest Hide and Seek task scores. Spatial Assembly refers to pretest Test of Spatial Assembly Scores. Numeracy refers to posttest Preschool Early Numeracy Screener scores. We included pretest numeracy, intervention condition, age, sex, race/ethnicity, ELL status, and pretest vocabulary skill as controls for this model, but they are not shown for the sake of simplicity as they are not relevant to our main research questions.

Results of the mediation analysis for working memory skills showed that working memory did not significantly mediate the relationship between pretest spatial assembly skills and posttest numeracy skills (*β* = 0.01, CI [0.00, 0.01], *p* = 0.315) when accounting for pretest numeracy skill. The direct association between pretest spatial assembly and posttest numeracy was significant (*β* = 0.14, *p* = 0.021) when the mean of working memory skills across both waves was added as a mediator. For a visualization of these results, please see Figure 1.

#### 3.2.2. Model 1b—Inhibitory Control

**Figure 2:**
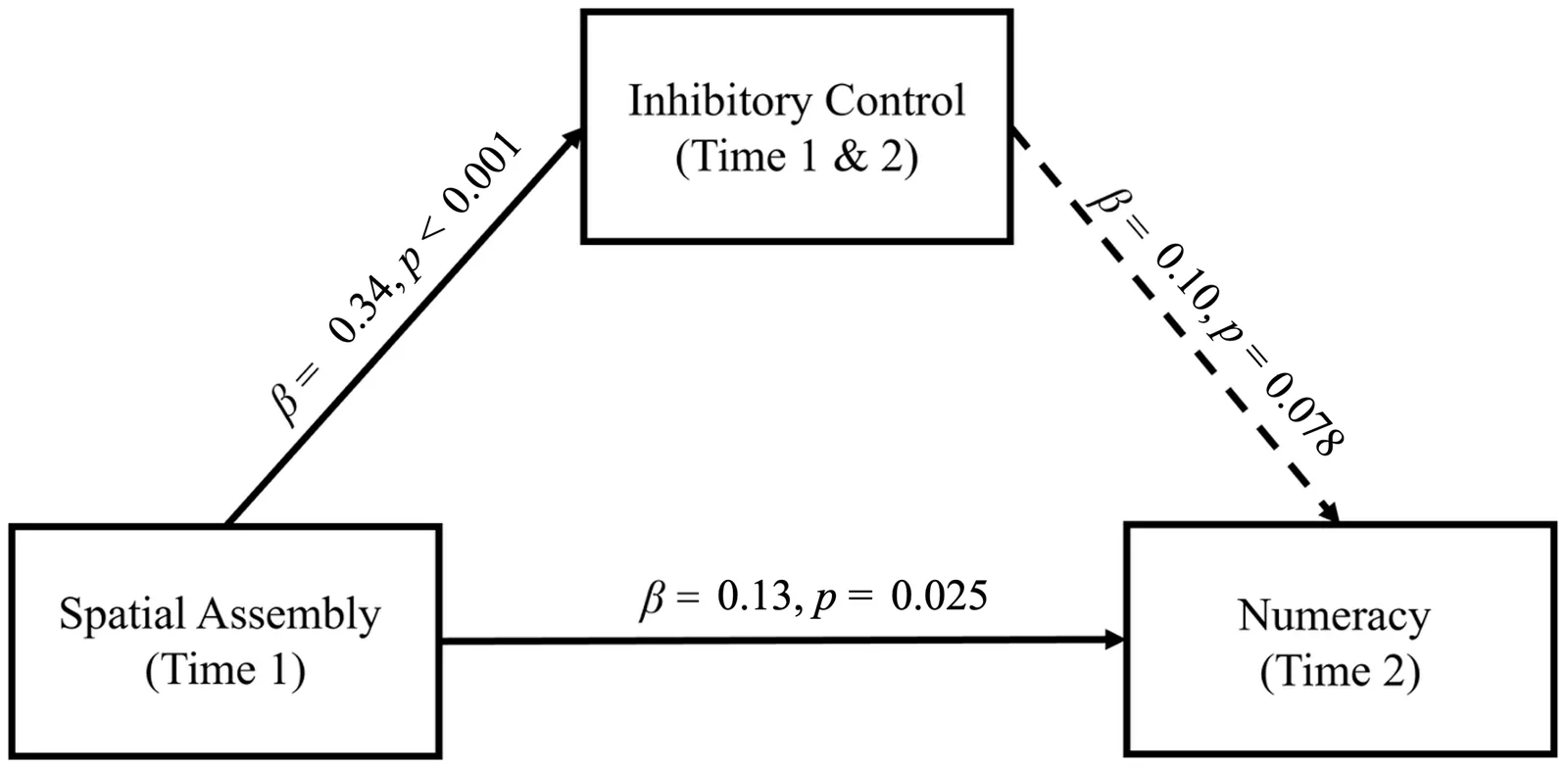
Inhibitory Control as a Mediator of the Spatial-Math Link. *Note.* Inhibitory Control refers to a mean score across pre- and posttest Day-Night Stroop task scores. Spatial Assembly refers to pretest Test of Spatial Assembly Scores. Numeracy refers to posttest Preschool Early Numeracy Screener scores. We included pretest numeracy, intervention condition, age, sex, race/ethnicity, ELL status, and pretest vocabulary skill as controls for this model, but they are not shown for the sake of simplicity as they are not relevant to our main research questions.

Results of the mediation analysis for inhibitory control skills showed that inhibitory control did not significantly mediate the relationship between pretest spatial assembly skills and posttest numeracy skills (*β* = 0.01, CI [0.00, 0.03], *p* = 0.097) when accounting for pretest numeracy skill. The direct association between pretest spatial assembly and posttest numeracy was significant (*β* = 0.13, *p* = 0.025) when the mean of inhibitory control skills across both waves was added as a mediator. For a visualization of these results, please see Figure 2.

#### 3.2.3. Model 1c—Cognitive Flexibility

**Figure 3:**
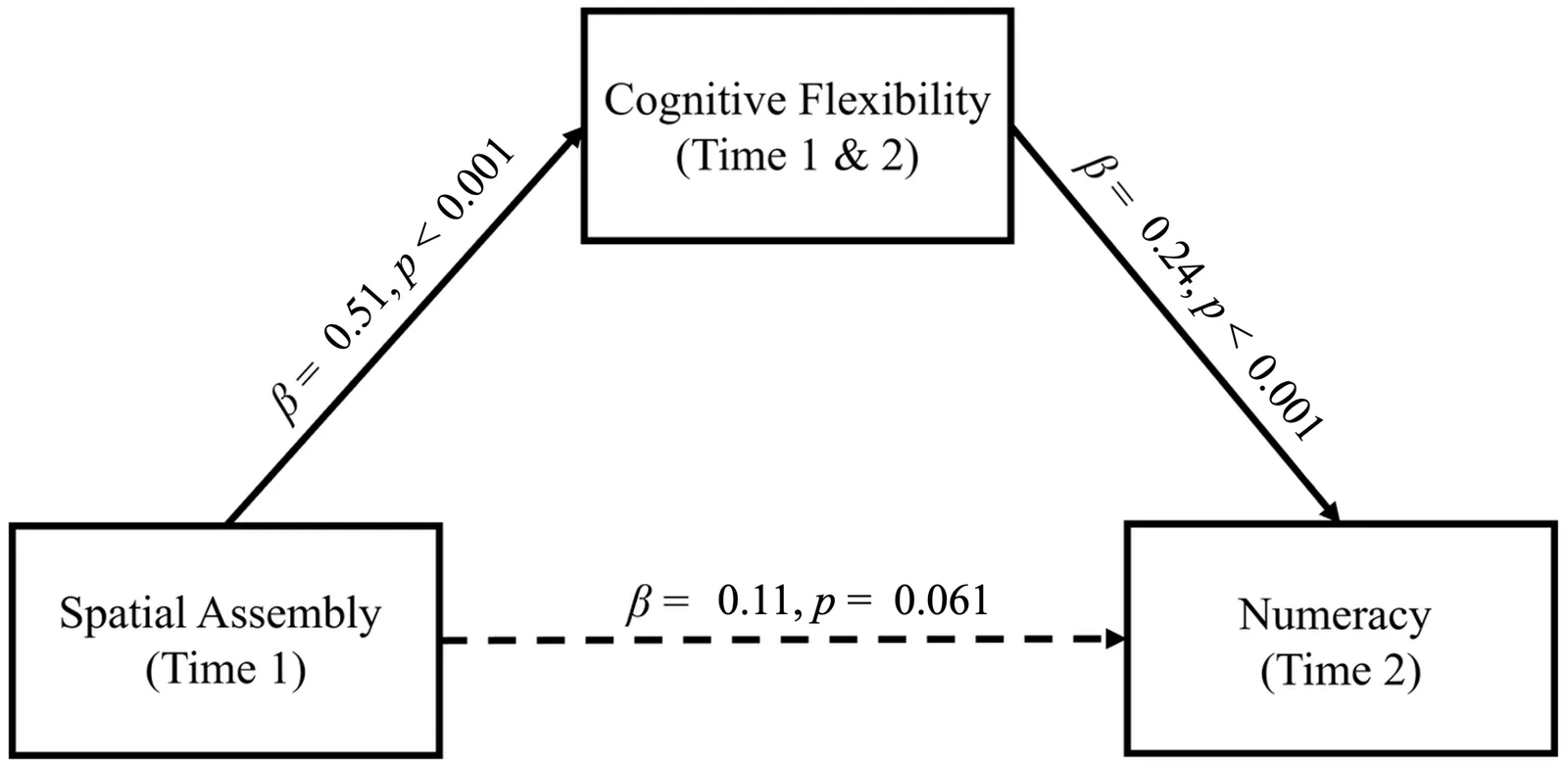
Cognitive Flexibility as a Mediator of the Spatial-Math Link. *Note.* Cognitive Flexibility refers to a mean score across pre- and posttest Card Sort task scores. Spatial Assembly refers to pretest Test of Spatial Assembly Scores. Numeracy refers to posttest Preschool Early Numeracy Screener scores. We included pretest numeracy, intervention condition, age, sex, race/ethnicity, ELL status, and pretest vocabulary skill as controls for this model, but they are not shown for the sake of simplicity as they are not relevant to our main research questions.

Results of the mediation analysis for cognitive flexibility skills showed that cognitive flexibility fully mediated the relationship between pretest spatial assembly skills and posttest numeracy skills (*β* = 0.05, CI [0.03, 0.08], *p* < 0.001) even when accounting for pretest numeracy skill. Specifically, the direct association between pretest spatial assembly and posttest numeracy became non-significant (*β* = 0.11, *p* = 0.061) when the mean of cognitive flexibility skills across both waves was added as a mediator. For a visualization of these results, please see Figure 3.

#### 3.2.4. Model 1d—Behavioral Self-Regulation

**Figure 4:**
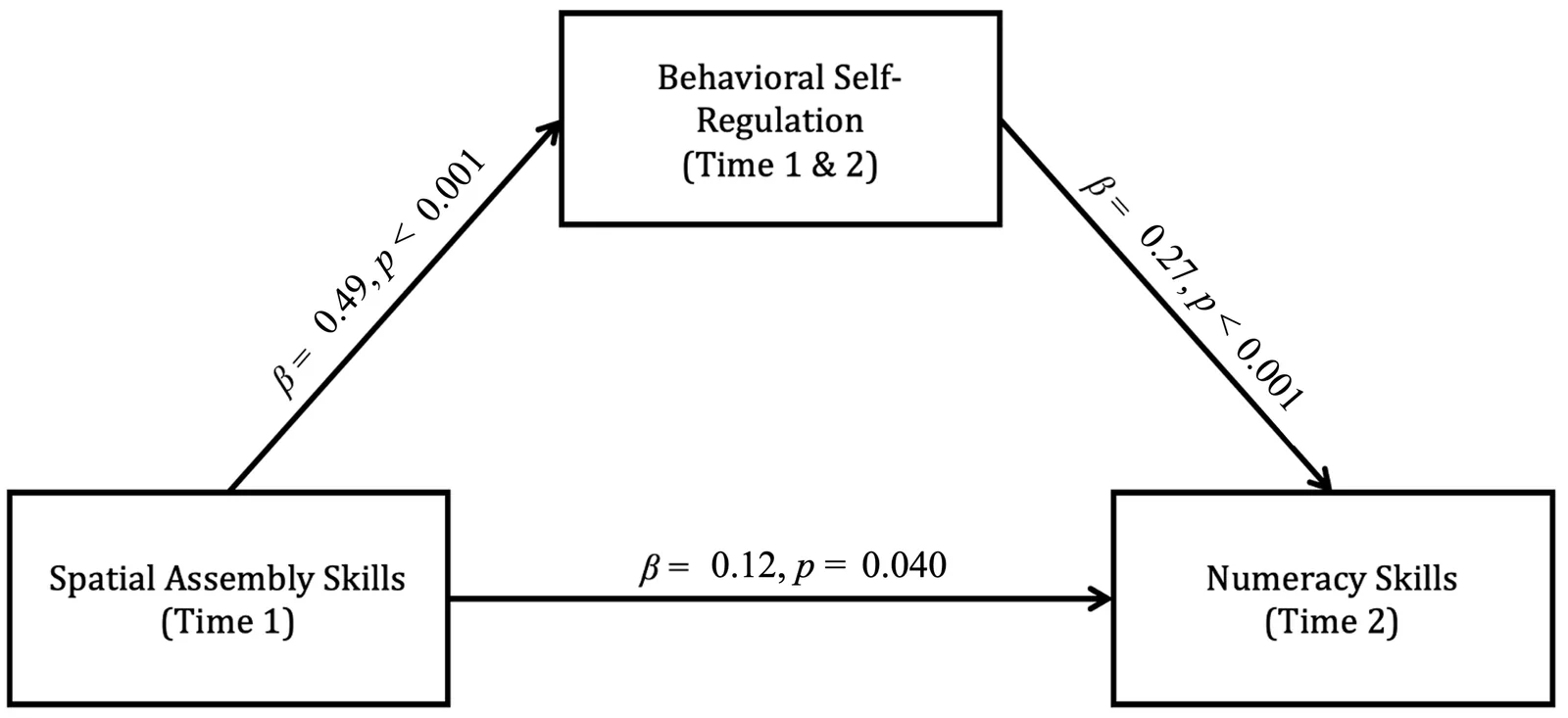
Behavioral Self-Regulation as a Mediator of the Spatial-Math Link. *Note.* Behavioral Self-Regulation refers to a mean score across pre- and posttest Head-Toes-Knees-Shoulders task scores. Spatial Assembly refers to pretest Test of Spatial Assembly Scores. Numeracy refers to posttest Preschool Early Numeracy Screener scores. We included pretest numeracy, intervention condition, age, sex, race/ethnicity, ELL status, and pretest vocabulary skill as controls for this model, but they are not shown for the sake of simplicity as they are not relevant to our main research questions.

Results of the mediation analysis for behavioral self-regulation skills showed that behavioral self-regulation partially mediated the relationship between pretest spatial assembly skills and posttest numeracy skills (*β* = 0.06, CI [0.03, 0.08], *p* < 0.001) even when accounting for pretest numeracy skill. The direct association between pretest spatial assembly and posttest numeracy remained significant (*β* = 0.12, *p* = 0.04) when the mean of behavioral self-regulation skills across both waves was added as a mediator. For a visualization of these results, please see Figure 4.

#### 3.2.5. Model 1e—Planning

**Figure 5:**
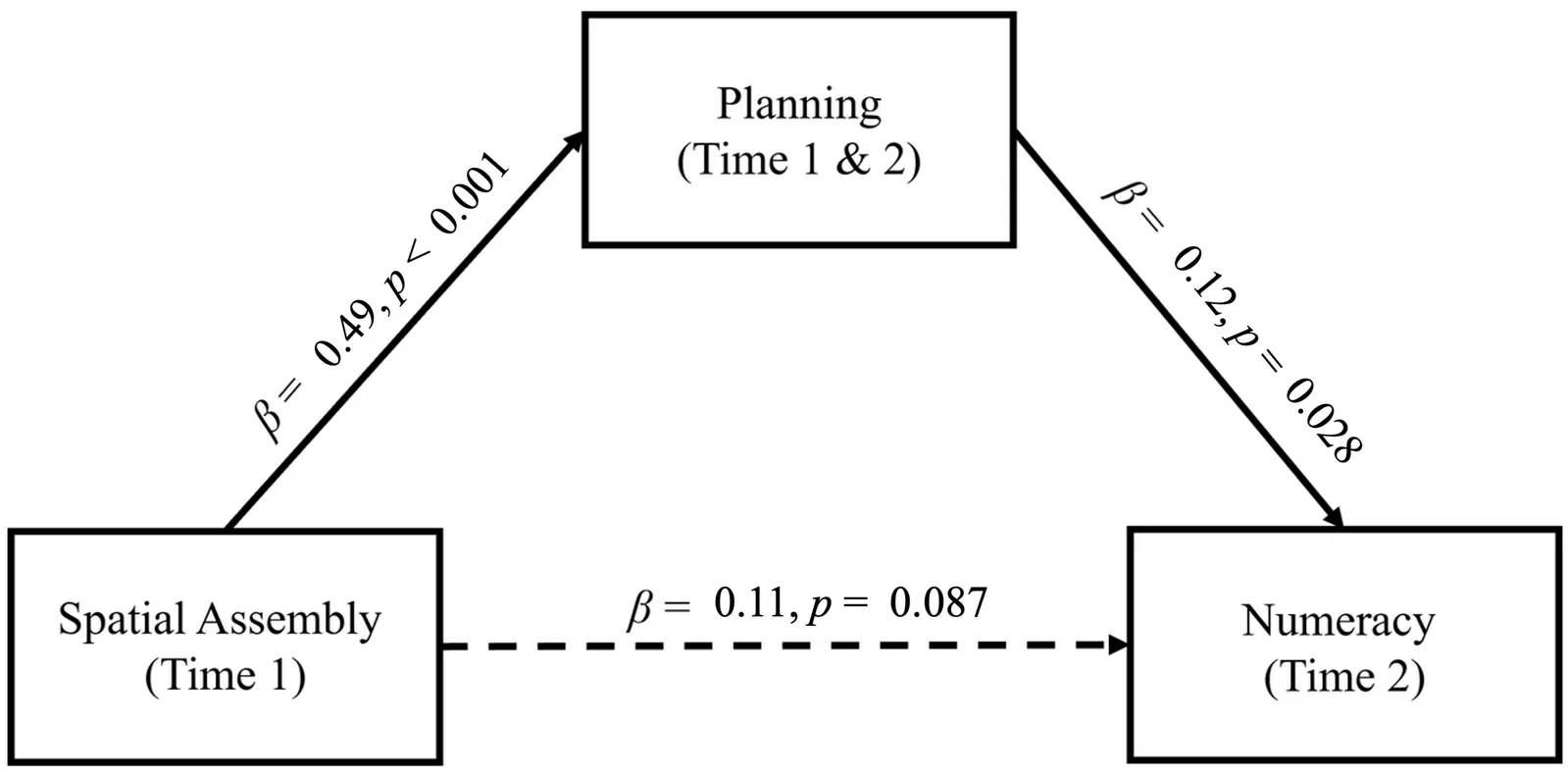
Planning as a Mediator of the Spatial-Math Link. *Note.* Planning refers to a mean score across pre- and posttest Tower of Hanoi task scores. Spatial Assembly refers to pretest Test of Spatial Assembly Scores. Numeracy refers to posttest Preschool Early Numeracy Screener scores. We included pretest numeracy, intervention condition, age, sex, race/ethnicity, ELL status, and pretest vocabulary skill as controls for this model, but they are not shown for the sake of simplicity as they are not relevant to our main research questions.

Results of the mediation analysis for planning skills showed that planning skills fully mediated the relationship between pretest spatial assembly skills and posttest numeracy skills (*β* = 0.03, CI [0.01, 0.05], *p* = 0.034) even when accounting for pretest numeracy skill. The direct association between pretest spatial assembly and posttest numeracy became non-significant (*β* = 0.11, *p* = 0.087) when mean planning skills across both waves were added as a mediator. For a visualization of these results, please see Figure 5.

#### 3.2.6. Model 2—Cognitive Flexibility, Behavioral Self-Regulation, and Planning as Mediators

**Figure 6:**
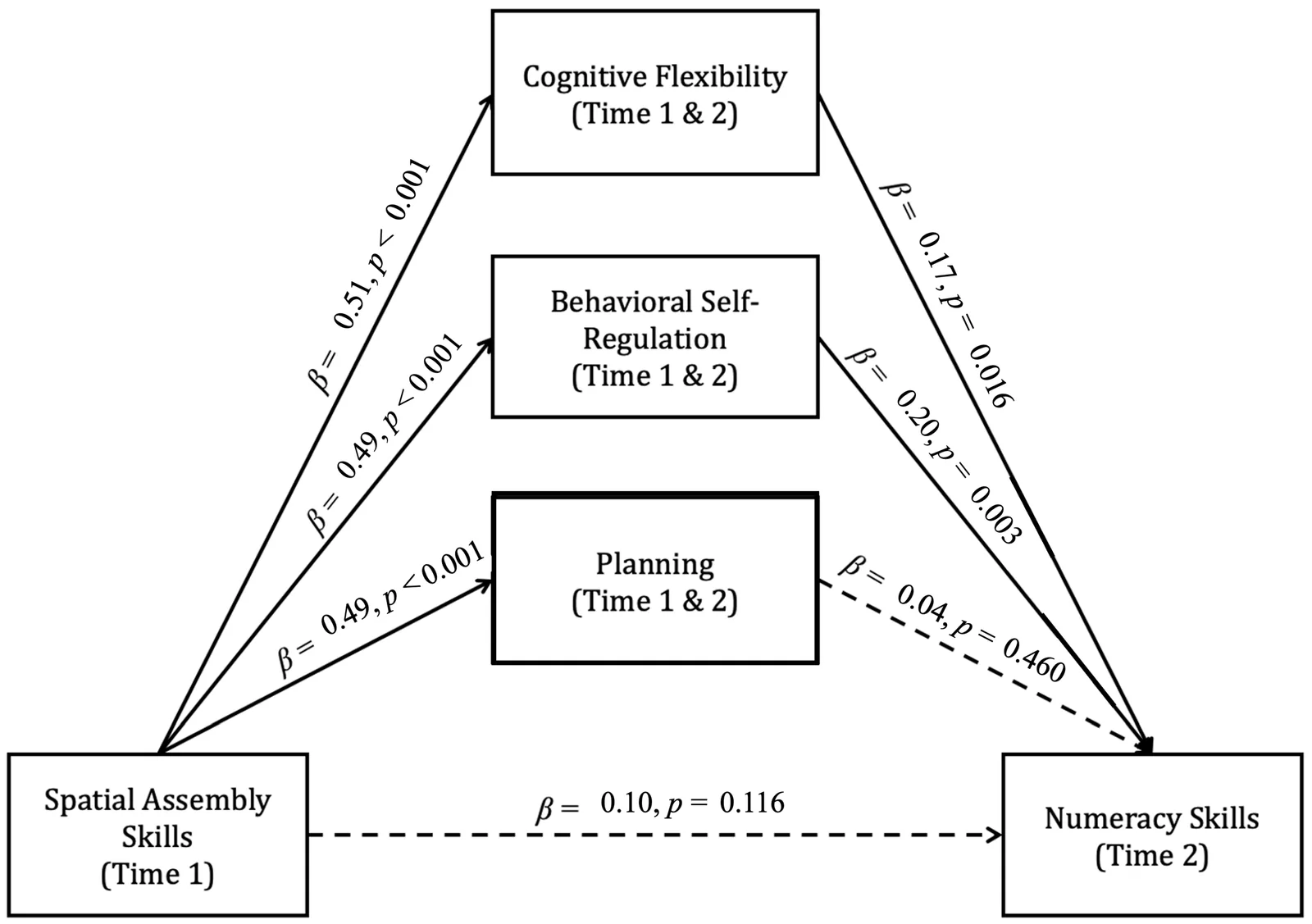
Cognitive Flexibility, Behavioral Self-Regulation, and Planning as Mediators. *Note.* Cognitive Flexibility refers to a mean score across pre- and posttest Card Sort task scores. Behavioral Self-Regulation refers to a mean score across pre- and posttest Head-Toes-Knees-Shoulders task scores. Planning refers to a mean score across pre- and posttest Tower of Hanoi task scores. Spatial Assembly refers to pretest Test of Spatial Assembly Scores. Numeracy refers to posttest Preschool Early Numeracy Screener scores. We included pretest numeracy, intervention condition, age, sex, race/ethnicity, ELL status, and pretest vocabulary skill as controls for this model, but they are not shown for the sake of simplicity as they are not relevant to our main research questions.

Results of our individual mediation analyses showed that cognitive flexibility, behavioral self-regulation, and planning emerged as significant mediators of the spatial-math link. To examine and understand unique associations between each of these executive function skills and the spatial-math link, we used a path analysis model that included all three of these executive function skills as mediators of the connection between spatial and math skills. Results of this analysis demonstrated that cognitive flexibility (*β* = 0.04, CI [0.01, 0.06], *p* = 0.020) and behavioral self-regulation (*β* = 0.04, CI [0.01, 0.07], *p* = 0.006) emerged as significant mediators, with the mediation effect of planning becoming non-significant (*β* = 0.01, CI [−0.02, 0.04], *p* = 0.462). The direct association between pretest spatial assembly and posttest numeracy became non-significant (*β* = 0.10, *p* = 0.116) when mean cognitive flexibility, behavioral self-regulation, and planning skills across both waves are added as mediators. For a visualization of these results, please see Figure 6.

## 4. Discussion

In the present study, we found preliminary evidence that suggests specific executive function skills may underlie the relationship between spatial and math skills in young children. Specifically, we tested whether various indicators of executive function (i.e., working memory, inhibitory control, cognitive flexibility, behavioral self-regulation, planning) act as mechanisms underlying the well-established spatial-math link. Our findings suggest that, when tested in separate models, cognitive flexibility, behavioral self-regulation, and planning are all mediators of the spatial-math link. However, when we ran a model with all three of these skills as mediators, cognitive flexibility and behavioral self-regulation remained significant mediators while planning no longer significantly explained any part of the spatial-math link. Importantly, our results provide preliminary evidence that individual executive function skills may underlie the connection between spatial and math skills in preschoolers.

### 4.1. Executive Function as a Mediator

Research has long established the links between spatial and math skills (for a meta-analysis, Atit et al., 2022), spatial and executive function skills (Garcia et al., 2022; Geer et al., 2025; Hawes et al., 2019; He et al., 2019; Kahl et al., 2022), and executive function and math skills (Mazzocco & Kover, 2007; Ribner et al., 2017; Schmitt et al., 2017) separately. However, only recently has some research considered the potential overlap between all three of these skills (Atit et al., 2022; Fung et al., 2020; Geer et al., 2025; Hawes et al., 2019; Kahl et al., 2021, 2022; Verdine et al., 2014b; Yang et al., 2019). Of this research, only one study has considered the potential that executive function mediates the link between spatial and math skills (Hawes et al., 2019), and results from this study indicated that a latent variable comprised of behavioral self-regulation and visuo-spatial working memory did not mediate the association between spatial skills and math. Although this study provides important insight to the field, this study did not examine behavioral self-regulation or visuo-spatial memory as separate mediators, nor did it include other executive function indicators in its analyses. While their decision to examine executive function as a unidimensional construct aligns with existing theories of the developmental trajectories of executive function (Ibbotson, 2023), it is possible that their results might have been different had they examined their executive functions individually. This is especially relevant as work with young children has shown that there is a differential link between distinct executive functions and various math skills (Purpura et al., 2017).

To expand upon the possibility that different executive functions may relate differently to math skills in preschoolers, we assessed whether five specific executive function skills (e.g., working memory, inhibitory control, cognitive flexibility, behavioral self-regulation, and planning) served as mediators of the spatial-math link across two time points. In contrast to the findings from Hawes et al. (2019), we found that three types of executive function separately mediated the spatial-math link. Specifically, in line with our expectations based on existing work that has shown that spatial skills predict cognitive flexibility (Kahl et al., 2022) and cognitive flexibility predicts math (Geer et al., 2025; Magalhães et al., 2020), we found that cognitive flexibility fully mediated the spatial-math link. We also found that planning significantly mediates the relationship between spatial and math skills. Additionally, we found that behavioral self-regulation partially mediates the spatial-math link. These results align closely with the abundance of literature that suggests these three skillsets (spatial, math, and executive function) are highly interconnected. More specifically, this work aligns theoretically with the arguments found in Hawes et al. (2019) paper that spatial and executive function are unique but highly intertwined skillsets that are both important to mathematical development. Our results suggest that, at some level, cognitive flexibility, behavioral self-regulation, and planning skills might explain the relationship between spatial and math skills. However, based on the extensive literature examining the spatial-math link, it is clear that spatial skills are still uniquely important to mathematical development; however, the mechanisms underlying the link between spatial and math skills remain unclear.

Although there is no other work examining these individual executive functions as mediators of the spatial-math link, there are practical explanations for why these executive functions (cognitive flexibility, behavioral self-regulation, and planning) may underlie the spatial-math association. On one hand, these three skills are the most complex of the executive function skills that we measured, which means that these measures capture executive functions that have higher cognitive load demands. In turn, this may suggest that children who perform better on these more complex executive function tasks may have an easier time with other cognitive tasks (such as spatial and math tasks) because they can handle tasks that demand more of them cognitively. It is also possible that engaging in spatial thought specifically for a spatial assembly task like the one included engages the same types of cognitive load that these specific executive functions do. For example, the TOSA engages children’s cognitive flexibility in that the task demands that participants easily swap from thinking and reasoning in two dimensions to thinking and reasoning in three dimensions. Similarly, the TOSA engages children’s behavioral self-regulation because this task requires children to have the ability to regulate the desired behavior of playing with the manipulatives (i.e., LEGO^®^ bricks) to complete the task at hand. Lastly, the TOSA engages children’s planning skills because the task itself demands that children take the goal (i.e., to recreate the model they are shown) and identify and execute the necessary steps to achieve said goal.

We did not find evidence that either working memory or inhibitory control mediated the spatial-math link. This is in line with the findings from Hawes et al. (2019), as they found a latent factor of executive function (which was comprised of three tasks, two of which were working memory tasks) was not a mediator of the association between spatial and math skills. However, our findings regarding working memory should be interpreted carefully, as our measure was not correlated with any of our other executive function measures and appeared to have a ceiling effect, suggesting it was too easy and may not have been accurately tapping into working memory skill. We also found no evidence that inhibitory control mediates the spatial-math link in our sample, although this may be reflective of the *types* of skills we measured. Neither the spatial assembly measure nor the numeracy skills measure necessarily required the ability to repress an incorrect response in lieu of a more acceptable response; therefore, it is possible that this mediation was non-significant because these particular spatial and math skills are not conceptually linked via the skills required by inhibitory control.

Based upon the results of our individual mediation models, we conducted a path analysis model that includes all three of the significant mediators from our first set of models (i.e., cognitive flexibility, behavioral self-regulation, and planning). We did this to examine the unique associations between each of these executive function skills and the spatial-math link. In this model, we found cognitive flexibility and behavioral self-regulation as mediators of the spatial-math link, with planning as a mediator, becoming non-significant. This finding preliminarily suggests that cognitive flexibility and behavioral self-regulation are key components that underlie the link between spatial and math skills in early childhood. Future research is needed to continue teasing apart the nature of these relations, particularly experimental research that would allow for the identification of causal links between these factors. Alternatively, future research with more than two timepoints would allow for more advanced statistical models (such as random-intercepts cross-lagged panel models) that would allow researchers to examine the directionality of the links between these factors over time.

### 4.2. Limitations and Future Directions

As with all research, the present study is not without limitations that should be acknowledged as we consider the results. The first limitation is that we only have two time-points of data, and we used an average for each executive function (the average of pre- and posttest scores), which does limit the causal conclusions that we can draw about our analyses as well as the types of analyses that we run. It is necessary to collect data on spatial, executive function, and math skills across additional waves of data in order to be able to determine the true causal nature of these links and perhaps conduct more complex models such as a random-intercepts cross-lagged panel model (RI-CLPM). In particular, the RI-CLPM model would account for between-child stable individual differences over time in these skills and allow us to partial out between-child stable differences from within-child process changes, similar to work done on the spatial-math link in the past (Bailey, 2017; Geer et al., 2019). Additionally, due to the fact that our study only has two time points, we are unable to definitively establish temporal ordering or developmental mechanisms. Thus, we must be cautious in our interpretation of the present findings to avoid making strong causal claims. However, the present study does set the stage for future research with additional time points to establish whether or not executive function serves as a causal mediator of the spatial-math link.

Another limitation is that the current dataset was pulled from a larger intervention study and, specifically, time point one for this study was the pretest data, and time point two was the posttest data. However, we did account for any intervention effects by controlling for condition; therefore, the intervention itself would have had strong impacts on the current findings. Regardless, future research should aim to examine if executive function mediates the spatial-math link in a study that does not involve any intervention to confirm that this mediation exists.

An additional limitation is that analyses were conducted using observed rather than latent variables. Although this approach is common in developmental research, it does not explicitly account for measurement error, which may attenuate observed associations. Future research using latent variable modeling could provide more precise estimates of the relationships among spatial skills, executive functions, and mathematics. We also did not evaluate measurement invariance across ELL and non-ELL children, because this was only included as a covariate and not as a moderator variable. Consequently, it cannot be determined whether all measures assessed the same underlying constructs in an identical manner across language groups. Future studies with a larger proportion of ELL children should examine measurement invariance before drawing strong conclusions regarding cross-group comparability.

Additionally, although our findings regarding working memory are in line with extant research (Hawes et al., 2019), it is important to note that our findings have been partially influenced by the fact that our measure of working memory had somewhat of a ceiling effect. Specifically, the total score on the task was 16, and the mean score for this task was more than 13.5 points for both pre- and posttest, meaning that the total score fell within one standard deviation of our sample means at both time points. Furthermore, our measure of working memory was not significantly correlated with our other measures of executive function; therefore, this measure simply may not be adequately measuring working memory in our sample. This lack of association between working memory and the other executive function measures could also possibly reflect the developing nature of executive function in preschool, where the structure of executive functions is still emerging and individual tasks often capture distinct cognitive demands in addition to executive processes, resulting in weaker or inconsistent relationships among components (Garon et al., 2008; Best & Miller, 2010). Due to the simplicity of the task and the potential ceiling effects, as well as the fact that our working memory task was not correlated with other executive function measures, we suggest that the results regarding working memory in this study should be taken lightly, and future work should aim to confirm whether working memory mediates the spatial-math link.

Another possible limitation, or rather something to keep in mind when interpreting our results, is that there is a possibility of overlapping task demands that may contribute to the relationships seen amongst variables. Specifically, our spatial measures task does share the same basic task demands as the planning task we used (i.e., presenting the child with a model and asking them to use their manipulatives to recreate the model). It is difficult to tease apart whether the current findings are based on the embedded nature of various executive functions within the included spatial and math measures, but future work could aim to tease this apart by including additional measures of spatial and math skills.

Lastly, our sample is exclusively from lower socioeconomic status backgrounds and, as such, these results may not be broadly generalizable. Future work should aim to address the nature of these relationships in various samples to determine if they remain statistically significant.

## 5. Conclusions

We found that the well-established spatial-math link is mediated by three specific executive function skills (i.e., cognitive flexibility, behavioral self-regulation, and planning). When examined together in one model, cognitive flexibility and behavioral self-regulation fully mediated the spatial-math link while planning became a non-significant mediator. This study provides preliminary evidence that executive function is a significant mediator of the link between spatial and math skills. Taken together with the extant research on the interplay between spatial, executive function, and math skills, our findings support the contention that learning in math is deeply rooted in both spatial cognition and executive function. This work sets the stage for future research that is needed to tease apart these relations, particularly the causal directions of these relationships in young children. This study represents a critical step in highlighting the importance of both spatial and executive function skills, in tandem, for establishing the domain-general foundational knowledge that is needed for the successful development of mathematics.

## Figures and Tables

**Table 1:** Descriptive Statistics for All Included Measures.

Variable	N	Minimum	Maximum	M	SD	Skew		Kurtosis	
						Statistic	S.E.	Statistic	S.E.
Pretest									
TOSA	232	23	80	52.16	13.21	0.02	0.16	−0.80	0.31
PENS	234	0	26	10.12	5.31	0.43	0.16	0.01	0.31
HAS	232	0	16	13.53	3.28	−1.65	0.16	2.26	0.31
DNS	234	0	32	19.76	9.52	−0.44	0.16	−0.80	0.31
Card Sort	239	2	23	10.05	5.67	0.96	0.16	−0.59	0.31
HTKS	238	0	58	7.37	13.70	1.98	0.16	2.87	0.31
TOH	232	0	6	1.99	1.77	0.92	0.16	0.05	0.31
PVT	232	−10.92	2.04	−5.62	2.31	0.04	0.16	−0.10	0.31
Posttest									
TOSA	210	23	81	59.34	12.76	−0.36	0.17	−0.60	0.33
PENS	213	0	26	12.71	5.98	0.06	0.17	−0.66	0.33
HAS	212	2	16	13.69	3.10	−1.57	0.17	1.82	0.33
DNS	211	0	32	24.05	8.81	−1.19	0.17	−0.38	0.33
Card Sort	213	0	23	12.49	6.65	0.21	0.17	−1.67	0.33
HTKS	210	0	57	14.30	17.95	0.92	0.17	−0.59	0.33
TOH	211	0	6	2.46	1.90	0.67	0.17	−0.75	0.33
PVT	208	−13.48	−0.36	−4.90	2.33	−0.45	0.17	0.21	0.34

*Note.* TOSA = Test of Spatial Assembly, PENS = Preschool Early Numeracy Skills Assessment, HAS = Hide-and-Seek task, DNS = Day-Night Stroop Task. HTKS = Head-Toes-Knees-Shoulders task, TOH = Tower of Hanoi task, PVT = Picture Vocabulary task.

**Table 2:** Zero-order correlations between all included measures.

Pretest								Posttest						
	TOSA	PENS	HAS	DNS	Card Sort	HTKS	ToH	TOSA	PENS	HAS	DNS	Card Sort	HTKS	ToH
Pretest														
TOSA														
PENS	0.54 ***													
HAS	0.11	0.15 *												
DNS	0.31 ***	0.40 ***	0.09											
Card Sort	0.47 ***	0.55 ***	0.10	0.30 ***										
HTKS	0.36 ***	0.48 ***	0.13	0.27 ***	0.45 ***									
ToH	0.39 ***	0.42 ***	0.21 **	0.39 ***	0.42 ***	0.31 ***								
PVT	0.38 ***	0.36 ***	0.08	0.39 ***	0.39 ***	0.24 ***	0.26 ***							
Posttest														
TOSA	0.65 ***	0.40 ***	0.04	0.24 ***	0.38 ***	0.27 ***	0.27 ***							
PENS	0.56 ***	0.73 ***	0.11	0.36 ***	0.59 ***	0.47 ***	0.40 ***	0.49 ***						
HAS	0.19 **	0.24 ***	0.12	0.10	0.18 **	0.15 *	0.25 ***	0.13	0.29 ***					
DNS	0.23 ***	0.58 ***	0.09	0.51 ***	0.19 **	0.26 ***	0.19 **	0.27 ***	0.35 ***	0.25 ***				
Card Sort	0.46 ***	0.53 ***	0.15 *	0.42 ***	0.61 ***	0.48 ***	0.33 ***	0.40 ***	0.56 ***	0.21 **	0.33 ***			
HTKS	0.47 ***	0.58 ***	0.19 **	0.37 ***	0.56 ***	0.68 ***	0.48 ***	0.39 ***	0.64 ***	0.25 ***	0.37 ***	0.55 ***		
ToH	0.45 ***	0.34 ***	0.21 **	0.23 ***	0.32 ***	0.36 ***	0.42 ***	0.38 ***	0.41 ***	0.27 ***	0.31 ***	0.42 ***	0.43 ***	
PVT	0.32 ***	0.29 ***	0.14 *	0.35 ***	0.34 ***	0.30 ***	0.29 ***	0.31 ***	0.27 ***	0.29 ***	0.44 ***	0.37 ***	0.32 ***	0.37 ***

*Note.* * refers to *p* < 0.05, ** refers to *p* < 0.01, *** refers to *p* < 0.001; TOSA = Test of Spatial Assembly Skills; PENS = Preschool Early Numeracy Skills; HAS = Hide and Seek task; DNS = Day-Night Stroop task; Card Sort = the Card Sorting task; HTKS = Head-Toes-Knees-Shoulders task; ToH = Tower of Hanoi task. The gray boxes refer to posttest tasks.

**Table 3:** Results of Mediation Models 1a–1e.

	*β/b*	*S.E.*	*p*
Model 1a			
Pretest Spatial → Posttest Math (direct)	0.14	0.05	0.021
Pretest Spatial → Posttest Math (total)	0.07	0.03	0.014
Pretest Spatial → Mean Working Memory	0.22	0.07	0.001
Mean Working Memory → Posttest Math	0.05	0.05	0.291
Pretest Spatial → Mean Working Memory → Posttest Math (indirect)	0.01	0.01	0.315
Pretest Math → Posttest Math	0.52	0.05	<0.001
Intervention Condition → Posttest Math	0.05	0.04	0.249
Pretest Vocabulary → Posttest Math	0.18	0.07	0.007
Age at Pretest → Posttest Math	0.09	0.06	0.108
Sex → Posttest Math	0.02	0.05	0.655
English Language Learner → Posttest Math	−0.13	0.06	0.039
Parent Education → Posttest Math	0.04	0.05	0.435
Race → Posttest Math	0.08	0.05	0.097
Model 1b			
Pretest Spatial → Posttest Math (direct)	0.13	0.06	0.025
Pretest Spatial → Posttest Math (total)	0.07	0.03	0.007
Pretest Spatial → Mean Inhibitory Control	0.34	0.06	<0.001
Mean Inhibitory Control → Posttest Math	0.10	0.06	0.078
Pretest Spatial → Mean Inhibitory Control → Posttest Math (indirect)	0.01	0.01	0.097
Pretest Math → Posttest Math	0.51	0.06	<0.001
Intervention Condition → Posttest Math	0.05	0.05	0.287
Pretest Vocabulary → Posttest Math	0.16	0.07	0.021
Age at Pretest → Posttest Math	0.10	0.06	0.095
Sex → Posttest Math	0.02	0.05	0.670
English Language Learner → Posttest Math	−0.14	0.06	0.033
Parent Education → Posttest Math	0.05	0.05	0.302
Race → Posttest Math	0.08	0.05	0.107
Model 1c			
Pretest Spatial → Posttest Math (direct)	0.11	0.06	0.061
Pretest Spatial → Posttest Math (total)	0.10	0.03	<0.001
Pretest Spatial → Mean Cognitive Flexibility	0.51	0.05	<0.001
Mean Cognitive Flexibility → Posttest Math	0.24	0.06	<0.001
Pretest Spatial → Mean Cognitive Flexibility → Posttest Math (indirect)	0.05	0.02	<0.001
Pretest Math → Posttest Math	0.46	0.06	<0.001
Intervention Condition → Posttest Math	0.04	0.05	0.372
Pretest Vocabulary → Posttest Math	0.10	0.07	0.158
Age at Pretest → Posttest Math	0.10	0.06	0.087
Sex → Posttest Math	0.02	0.05	0.721
English Language Learner → Posttest Math	−0.08	0.07	0.218
Parent Education → Posttest Math	0.05	0.05	0.384
Race → Posttest Math	0.11	0.05	0.030
Model 1d			
Pretest Spatial → Posttest Math (direct)	0.12	0.06	0.040
Pretest Spatial → Posttest Math (total)	0.11	0.03	<0.001
Pretest Spatial → Mean Behavioral Self-Regulation	0.49	0.05	<0.001
Mean Behavioral Self-Regulation → Posttest Math	0.27	0.06	<0.001
Pretest Spatial → Mean Behavioral Self-Regulation → Posttest Math (indirect)	0.06	0.01	<0.001
Pretest Math → Posttest Math	0.45	0.06	<0.001
Intervention Condition → Posttest Math	0.06	0.04	0.191
Pretest Vocabulary → Posttest Math	0.16	0.07	0.022
Age at Pretest → Posttest Math	0.05	0.06	0.394
Sex → Posttest Math	0.02	0.05	0.747
English Language Learner → Posttest Math	−0.10	0.06	0.132
Parent Education → Posttest Math	0.02	0.05	0.698
Race → Posttest Math	0.09	0.05	0.079
Model 1e			
Pretest Spatial → Posttest Math (direct)	0.11	0.06	0.087
Pretest Spatial → Posttest Math (total)	0.07	0.03	0.007
Pretest Spatial → Mean Behavioral Self-Regulation	0.49	0.05	<0.001
Mean Behavioral Self-Regulation → Posttest Math	0.12	0.06	0.028
Pretest Spatial → Mean Behavioral Self-Regulation → Posttest Math (indirect)	0.03	0.01	0.034
Pretest Math → Posttest Math	0.51	0.05	<0.001
Intervention Condition → Posttest Math	0.06	0.04	0.204
Pretest Vocabulary → Posttest Math	0.16	0.07	0.025
Age at Pretest → Posttest Math	0.10	0.06	0.079
Sex → Posttest Math	0.03	0.05	0.561
English Language Learner → Posttest Math	−0.12	0.06	0.065
Parent Education → Posttest Math	0.05	0.05	0.305
Race → Posttest Math	0.09	0.05	0.086

*Note*. Model 1a has working memory as the mediator. Model 1b has inhibitory control as the mediator. Model 1c has cognitive flexibility as the mediator. Model 1d has behavioral self-regulation as the mediator. Model 1e has planning as the mediator.

**Table 4:** Results of Mediation Model 2.

	*β/b*	*S.E.*	*p*
Pretest Spatial → Posttest Math (direct)	0.10	0.06	0.116
Pretest Spatial → Mean Cognitive Flexibility	0.17	0.07	<0.001
Mean Cognitive Flexibility → Posttest Math	0.51	0.05	0.016
Pretest Spatial → Mean Behavioral Self-Regulation	0.20	0.07	<0.001
Mean Behavioral Self-Regulation → Posttest Math	0.49	0.05	0.003
Pretest Spatial → Mean Planning	0.49	0.05	<0.001
Mean Planning → Posttest Math	0.04	0.06	0.460
Pretest Spatial → Mean Cognitive Flexibility → Posttest Math (indirect, ab)	0.04	0.02	0.006
Pretest Spatial → Mean Behavioral Self-Regulation → Posttest Math (indirect, cd)	0.04	0.02	0.020
Pretest Spatial → Mean Planning → Posttest Math (indirect, ef)	0.01	0.01	0.462
Pretest Spatial → Posttest Math (total, ab)	0.08	0.03	0.007
Pretest Spatial → Posttest Math (total, cd)	0.08	0.03	0.009
Pretest Spatial → Posttest Math (total, ef)	0.05	0.03	0.055
Pretest Math → Posttest Math	0.42	0.06	<0.001
Intervention Condition → Posttest Math	0.05	0.05	0.273
Pretest Vocabulary → Posttest Math	0.10	0.07	0.180
Age at Pretest → Posttest Math	0.07	0.06	0.275
Sex → Posttest Math	0.01	0.05	0.829
English Language Learner → Posttest Math	−0.07	0.07	0.287
Parent Education → Posttest Math	0.03	0.05	0.560
Race → Posttest Math	0.10	0.05	0.039

*Note.* The indicator ab refers to the mediation pathway through cognitive flexibility. The indicator cd refers to the mediation pathway through behavioral self-regulation. The indicator ef refers to the mediation pathway through planning.

## Data Availability

The datasets presented in this article are not readily available because the dataset is part of a larger ongoing study and we do not have permission to share it at this time. Requests to access the data should be directed to Dr. Sara A. Schmitt.

## References

[B1-behavsci-16-01545] Acock A. C. (2013). Discovering structural equation modeling using Stata. Stata press books.

[B2-behavsci-16-01545] Allain P., Nicoleau S., Pinon K., Etcharry-Bouyx F., Barré J., Berrut G., Dubas F., Le Gall D. (2005). Executive functioning in normal aging: A study of action planning using the Zoo Map Test. Brain and Cognition.

[B3-behavsci-16-01545] Atit K., Power J. R., Pigott T., Lee J., Geer E. A., Uttal D. H., Ganley C. M., Sorby S. A. (2022). Examining the relations between spatial skills and mathematical performance: A meta-analysis. Psychonomic Bulletin & Review.

[B4-behavsci-16-01545] Aunola K., Leskinen E., Lerkkanen M. K., Nurmi J. E. (2004). Developmental dynamics of math performance from preschool to grade 2. Journal of Educational Psychology.

[B5-behavsci-16-01545] Bailey D. H. (2017). Causal inference and the spatial–math link in early childhood. Monographs of the Society for Research in Child Development.

[B6-behavsci-16-01545] Baroody A. J., Gannon K. E., Berent R., Ginsburg H. P. (1984). The development of basic formal mathematics abilities. Acta Paedologica.

[B7-behavsci-16-01545] Best J. R., Miller P. H. (2010). A developmental perspective on executive function. Child Development.

[B8-behavsci-16-01545] Carlson S. M., Moses L. J., Claxton L. J. (2004). Individual differences in executive functioning and theory of mind: An investigation of inhibitory control and planning ability. Journal of Experimental Child Psychology.

[B9-behavsci-16-01545] Casey M. B., Nutall R. L., Pezaris E. (2001). Spatial-mechanical reasoning skills versus mathematics self-confidence as mediators of gender differences on mathematics subtests using cross-national gender-based items. Journal for Research in Mathematics Education.

[B10-behavsci-16-01545] Casey M. B., Nuttall R., Pezaris E., Benbow C. P. (1995). The influence of spatial ability on gender differences in mathematics college entrance test scores across diverse samples. Developmental Psychology.

[B11-behavsci-16-01545] Claessens A., Engel M. (2013). How important is where you start? Early mathematics knowledge and later school success. Teachers College Record.

[B12-behavsci-16-01545] Cole D. A., Maxwell S. E. (2003). Testing mediational models with longitudinal data: Questions and tips in the use of structural equation modeling. Journal of Abnormal Psychology.

[B13-behavsci-16-01545] Delgado A. R., Prieto G. (2004). Cognitive mediators and sex-related differences in mathematics. Intelligence.

[B14-behavsci-16-01545] Diamond A. (2013). Executive functions. Annual Review of Psychology.

[B15-behavsci-16-01545] Eason S. H., Ramani G. B. (2020). Parent–child math talk about fractions during formal learning and guided play activities. Child Development.

[B16-behavsci-16-01545] Ellis A., Ahmed S. F., Zeytinoglu S., Isbell E., Calkins S. D., Leerkes E. M., Grammer J. K., Gehring W. J., Morrison F. J., Davis-Kean P. E. (2021). Reciprocal associations between executive function and academic achievement: A conceptual replication of Schmitt et al. (2017). Journal of Numerical Cognition.

[B17-behavsci-16-01545] Fung W. K., Chung K. K. H., Lam C. B. (2020). Mathematics, executive functioning, and visual–spatial skills in Chinese kindergarten children: Examining the bidirectionality. Journal of Experimental Child Psychology.

[B18-behavsci-16-01545] Ganley C. M., Vasilyeva M. (2011). Sex differences in the relation between math performance, spatial skills, and attitudes. Journal of Applied Developmental Psychology.

[B19-behavsci-16-01545] Garcia N. L., Dick A. S., Pruden S. M. (2022). Contributions of executive function to spatial thinking in young children. Infant and Child Development.

[B20-behavsci-16-01545] Garon N., Bryson S. E., Smith I. M. (2008). Executive function in preschoolers: A review using an integrative framework. Psychological Bulletin.

[B21-behavsci-16-01545] Garon N., Smith I. M., Bryson S. E. (2014). A novel executive function battery for preschoolers: Sensitivity to age differences. Child Neuropsychology.

[B22-behavsci-16-01545] Geer E. A., Devlin B. L., Zehner T., Korucu I., Bryant L. M., Purpura D. J., Duncan R. J., Schmitt S. A. (2025). Does executive function moderate the spatial-math link in preschoolers?. Journal of Experimental Child Psychology.

[B23-behavsci-16-01545] Geer E. A., Quinn J. M., Ganley C. M. (2019). Relations between spatial skills and math performance in elementary school children: A longitudinal investigation. Developmental Psychology.

[B24-behavsci-16-01545] Gershon R. C., Fox R. S., Manly J. J., Mungas D. M., Nowinski C. J., Roney E. M., Slotkin J. (2020). The NIH toolbox: Overview of development for use with Hispanic populations. Journal of the International Neuropsychological Society.

[B25-behavsci-16-01545] Gerstadt C. L., Hong Y. J., Diamond A. (1994). The relationship between cognition and action: Performance of children 312–7 years old on a stroop-like day-night test. Cognition.

[B26-behavsci-16-01545] Hair J. F., Black W. C., Babin B. J., Anderson R. E., Tatham R. L. (2010). Multivariate data analysis: Global edition.

[B27-behavsci-16-01545] Halpern D. F., Benbow C. P., Geary D. C., Gur R. C., Hyde J. S., Gernsbacher M. A. (2007). The science of sex differences in science and mathematics. Psychological Science in the Public Interest.

[B28-behavsci-16-01545] Hawes Z., Moss J., Caswell B., Seo J., Ansari D. (2019). Relations between numerical, spatial, and executive function skills and mathematics achievement: A latent-variable approach. Cognitive Psychology.

[B29-behavsci-16-01545] He Z. H., Li B. H., Yin W. G. (2019). Executive function and mental rotation during middle childhood: The effect of age. The Journal of Genetic Psychology.

[B30-behavsci-16-01545] Hodes R. J., Insel T. R., Landis S. C. (2013). The NIH toolbox: Setting a standard for biomedical research. Neurology.

[B31-behavsci-16-01545] Ibbotson P. (2023). The development of executive function: Mechanisms of change and functional pressures. Journal of Cognition and Development.

[B32-behavsci-16-01545] Jones S. M., Bailey R., Barnes S. P., Partee A., Bailey R., Barnes S., Partee A. (2016). Executive function mapping project.

[B33-behavsci-16-01545] Jordan N. C., Kaplan D., Ramineni C., Locuniak M. N. (2009). Early math matters: Kindergarten number competence and later mathematics outcomes. Developmental Psychology.

[B34-behavsci-16-01545] Kahl T., Grob A., Segerer R., Möhring W. (2021). Executive functions and visual-spatial skills predict mathematical achievement: Asymmetrical associations across age. Psychological Research.

[B35-behavsci-16-01545] Kahl T., Segerer R., Grob A., Möhring W. (2022). Bidirectional associations among executive functions, visual-spatial skills, and mathematical achievement in primary school students: Insights from a longitudinal study. Cognitive Development.

[B36-behavsci-16-01545] King Y. A., Purpura D. J. (2021). Direct numeracy activities and early math skills: Math language as a mediator. Early Childhood Research Quarterly.

[B37-behavsci-16-01545] Lachance J. A., Mazzocco M. M. (2006). A longitudinal analysis of sex differences in math and spatial skills in primary school age children. Learning and Individual Differences.

[B38-behavsci-16-01545] Lezak M. (1995). Neuropsychological assessment.

[B39-behavsci-16-01545] Libertus M. E., Feigenson L., Halberda J. (2013). Numerical approximation abilities correlate with and predict informal but not formal mathematics abilities. Journal of Experimental Child Psychology.

[B40-behavsci-16-01545] Linn M. C., Petersen A. C. (1985). Emergence and characterization of sex differences in spatial ability: A meta-analysis. Child Development.

[B41-behavsci-16-01545] Magalhães S., Carneiro L., Limpo T., Filipe M. (2020). Executive functions predict literacy and mathematics achievements: The unique contribution of cognitive flexibility in grades 2, 4, and 6. Child Neuropsychology.

[B42-behavsci-16-01545] Mazzocco M. M., Kover S. T. (2007). A longitudinal assessment of executive function skills and their association with math performance. Child Neuropsychology.

[B43-behavsci-16-01545] McClelland M. M., Cameron C. E. (2011). Self-regulation and academic achievement in elementary school children. New Directions for Child and Adolescent Development.

[B44-behavsci-16-01545] Meece J. L., Parsons J. E., Kaczala C. M., Goff S. B. (1982). Sex differences in math achievement: Toward a model of academic choice. Psychological Bulletin.

[B45-behavsci-16-01545] Mesulam M. M. (2002). The human frontal lobes: Transcending the default mode through contingent encoding. Principles of Frontal Lobe Function.

[B46-behavsci-16-01545] Mix K. S., Levine S. C., Cheng Y. L., Young C., Hambrick D. Z., Ping R., Konstantopoulos S. (2016). Separate but correlated: The latent structure of space and mathematics across development. Journal of Experimental Psychology: General.

[B47-behavsci-16-01545] Miyake A., Friedman N. P., Rettinger D. A., Shah P., Hegarty M. (2001). How are visuospatial working memory, executive functioning, and spatial abilities related? A latent-variable analysis. Journal of Experimental Psychology: General.

[B48-behavsci-16-01545] Muthén L. K., Muthén B. O. (1998–2017). Mplus user’s guide.

[B49-behavsci-16-01545] Duncan G. J., National Institute of Child Health and Human Development Early Child Care Research Network (2003). Modeling the impacts of child care quality on children’s preschool cognitive development. Child Development.

[B50-behavsci-16-01545] Ponitz C. C., McClelland M. M., Matthews J. S., Morrison F. J. (2009). A structured observation of behavioral self-regulation and its contribution to kindergarten outcomes. Developmental Psychology.

[B51-behavsci-16-01545] Purpura D. J., Logan J. A., Hassinger-Das B., Napoli A. R. (2017). Why do early mathematics skills predict later reading? The role of mathematical language. Developmental Psychology.

[B52-behavsci-16-01545] Purpura D. J., Lonigan C. J. (2013). Informal numeracy skills: The structure and relations among numbering, relations, and arithmetic operations in preschool. American Educational Research Journal.

[B53-behavsci-16-01545] Purpura D. J., Reid E. E., Eiland M. D., Baroody A. J. (2015). Using a brief preschool early numeracy skills screener to identify young children with mathematics difficulties. School Psychology Review.

[B54-behavsci-16-01545] Ribner A. D., Willoughby M. T., Blair C. B., Family Life Project Key Investigators (2017). Executive function buffers the association between early math and later academic skills. Frontiers in Psychology.

[B55-behavsci-16-01545] Riegle-Crumb C., Grodsky E. (2010). Racial-ethnic differences at the intersection of math course-taking and achievement. Sociology of Education.

[B56-behavsci-16-01545] Rittle-Johnson B., Zippert E. L., Boice K. L. (2019). The roles of patterning and spatial skills in early mathematics development. Early Childhood Research Quarterly.

[B57-behavsci-16-01545] Schmitt S. A., Geldhof G. J., Purpura D. J., Duncan R., McClelland M. M. (2017). Examining the relations between executive function, math, and literacy during the transition to kindergarten: A multi-analytic approach. Journal of Educational Psychology.

[B58-behavsci-16-01545] Schmitt S. A., Korucu I., Napoli A. R., Bryant L. M., Purpura D. J. (2018). Using block play to enhance preschool children’s mathematics and executive functioning: A randomized controlled trial. Early Childhood Research Quarterly.

[B59-behavsci-16-01545] Schmitt S. A., Purpura D. J., Duncan R. J., Bryant L., Zehner T. M., Devlin B. L., Geer E. A., Paes T. A. (2025). Testing block play as an effective mechanism for promoting early math, executive function, and spatial skills in preschoolers from low-income backgrounds. Early Childhood Research Quarterly.

[B60-behavsci-16-01545] Shea D. L., Lubinski D., Benbow C. P. (2001). Importance of assessing spatial ability in intellectually talented young adolescents: A 20-year longitudinal study. Journal of Educational Psychology.

[B61-behavsci-16-01545] Simon H. A. (1975). The functional equivalence of problem solving skills. Cognitive Psychology.

[B62-behavsci-16-01545] Slusser E., Ribner A., Shusterman A. (2019). Language counts: Early language mediates the relationship between parent education and children’s math ability. Developmental Science.

[B63-behavsci-16-01545] StataCorp LLC (2017). STATA finite mixture models reference manual.

[B64-behavsci-16-01545] Ten Braak D., Lenes R., Purpura D. J., Schmitt S. A., Størksen I. (2022). Why do early mathematics skills predict later mathematics and reading achievement? The role of executive function. Journal of Experimental Child Psychology 214.

[B65-behavsci-16-01545] Uttal D. H., Miller D. I., Newcombe N. S. (2013). Exploring and enhancing spatial thinking: Links to achievement in science, technology, engineering, and mathematics?. Current Directions in Psychological Science.

[B66-behavsci-16-01545] Vaidya C. J., You X., Mostofsky S., Pereira F., Berl M. M., Kenworthy L. (2020). Data-driven identification of subtypes of executive function across typical development, attention deficit hyperactivity disorder, and autism spectrum disorders. Journal of Child Psychology and Psychiatry.

[B67-behavsci-16-01545] Vasilyeva M., Lourenco S. F., Overton W. F., Lerner R. M. (2010). Spatial development. The Handbook of life-span development, vol. 1. cognition, biology, and methods.

[B68-behavsci-16-01545] Verdine B. N., Golinkoff R. (2018). Test of Spatial Assembly (TOSA) instruction manual.

[B69-behavsci-16-01545] Verdine B. N., Golinkoff R. M., Hirsh-Pasek K., Newcombe N. S. (2017). I. Spatial skills, their development, and their links to mathematics. Monographs of the Society for Research in Child Development.

[B70-behavsci-16-01545] Verdine B. N., Golinkoff R. M., Hirsh-Pasek K., Newcombe N. S., Filipowicz A. T., Chang A. (2014a). Deconstructing building blocks: Preschoolers’ spatial assembly performance relates to early mathematical skills. Child Development.

[B71-behavsci-16-01545] Verdine B. N., Irwin C. M., Golinkoff R. M., Hirsh-Pasek K. (2014b). Contributions of executive function and spatial skills to preschool mathematics achievement. Journal of Experimental Child Psychology.

[B72-behavsci-16-01545] Walsh V. (2003). A theory of magnitude: Common cortical metrics of time, space and quantity. Trends in Cognitive Sciences.

[B73-behavsci-16-01545] Welsh M. C. (1991). Rule-guided behavior and self-monitoring on the Tower of Hanoi disk-transfer task. Cognitive Development.

[B74-behavsci-16-01545] Yang X., Chung K. K. H., McBride C. (2019). Longitudinal contributions of executive functioning and visual-spatial skills to mathematics learning in young Chinese children. Educational Psychology.

[B75-behavsci-16-01545] Zelazo P. D. (2006). The Dimensional Change Card Sort (DCCS): A method of assessing executive function in children. Nature Protocols.

[B76-behavsci-16-01545] Zelazo P. D., Müller U., Frye D., Marcovitch S., Argitis G., Boseovski J., Chiang J. K., Hongwanishkul D., Schuster B. V., Sutherland A., Carlson S. M. (2003). The development of executive function in early childhood. Monographs of the Society for Research in Child Development.

[B77-behavsci-16-01545] Zippert E. L., Eason S. H., Marshall S., Ramani G. B. (2019). Preschool children’s math exploration during play with peers. Journal of Applied Developmental Psychology.

